# Predictors of vocational status in schizophrenia patients – Results from the Polish nationwide survey

**DOI:** 10.1177/0020764015577841

**Published:** 2015-12

**Authors:** Andrzej Kiejna, Patryk Piotrowski, Błażej Misiak, Tomasz Adamowski, Agata Schubert, Iwona Skrzekowska-Baran, Dorota Frydecka

**Affiliations:** 1 Department of Psychiatry, Wroclaw Medical University, Wroclaw, Poland; 2 Department of Genetics, Wroclaw Medical University, Wroclaw, Poland; 3 Janssen-Cilag Polska, Warsaw, Poland

**Keywords:** Schizophrenia, vocational status, medical comorbidity, inpatient hospitalizations, nationwide survey

## Abstract

**Background:**

Steady employment constitutes one of most important aspects of functional recovery in schizophrenia. Therefore, there is a need for understanding clinical and demographic factors predicting vocational status in schizophrenia.

**Methods:**

Clinical and demographic data of 1,010 schizophrenia patients were gathered from public outpatient clinics. We compared patients who maintained employment between the diagnosis time point and the day of assessment, with the patients who were employed in the diagnosis time point but were unemployed on the day of assessment with respect to clinical and demographic variables.

**Results:**

Lower educational attainment, lower-income region of residence, medical comorbidities (obesity, diabetes and hypertension), first hospitalization at inpatient unit in comparison with the day hospital, higher total number of hospitalizations and the number of inpatient hospitalizations were found to serve as predictors of unemployment throughout the course of schizophrenia. After application of Bonferroni correction and logistic binary regression analysis, lower educational attainment, higher number of inpatient hospitalizations and obesity predicted unemployment.

**Conclusion:**

Education, obesity and the number of inpatient hospitalizations seem to predict vocational outcome in schizophrenia. This study warrants further investigation of medical comorbidities in schizophrenia in terms of social consequences in order to indicate the direction of this relationship.

## Introduction

Schizophrenia is a severe mental illness with chronic and relapsing course that is associated with functional disability. Although functional decline often occurs in schizophrenia patients, poor illness outcome is not an indispensable part of the disorder (Zipursky, Reilly, & Murray, 2013). It is of great importance that symptomatic remission is not synonymous with functional recovery, as it is more common than sufficient overall functioning (Harvey et al., 2012).

Steady employment constitutes one of the most important parts of functional recovery (Schennach-Wolff, Riedel, et al., 2012; Ucok, Gorwood, & Karadayi, 2012) and is associated with significant reductions in the cost of medical service use (Bush, Drake, Xie, McHugo, & Haslett, 2009) in schizophrenia patients. For instance, apart from symptomatic remission, cordial family relationships, maintenance of social relationships, independent living and involvement in work constitute the core components of most widely implemented operational criteria of functional recovery from schizophrenia (Liberman, Kopelowicz, Ventura, & Gutkind, 2002). Unemployment is one of the most important psychosocial disabilities experienced by schizophrenia patients (Switaj et al., 2012). Based on the recent systematic review, Marwaha and Johnson (Marwaha & Johnson, 2004) found that employment rates in schizophrenia patients are 8%–35% in European studies. In light of high prevalence of unemployment, previous studies have aimed at investigating factors that predict poor vocational status in this group of patients. Inconsistent results indicate that such demographic variables as gender (Beiser et al., 1994; Harrison, Croudace, Mason, Glazebrook, & Medley, 1996; McCreadie, 1982; Ran et al., 2011; Ucok et al., 2012), educational attainment (Cougnard, Goumilloux, Monello, & Verdoux, 2009), previous work history (Mueser, Salyers, & Mueser, 2001; Schennach-Wolff, Musil, Moller, & Riedel, 2012), marital status (Evert, Harvey, Trauer, & Herrman, 2003; Loganathan & Murthy, 2011; Midin et al., 2011; Srinivasan & Thara, 1997) and rural or urban environment (Munk-Jorgensen & Mortensen, 1992; Yang et al., 2013) are associated with vocational outcome. In turn, the following clinical factors have been found to increase unemployment rates: severity of negative symptoms (Erickson, Jaafari, & Lysaker, 2011; Marwaha et al., 2009; Schennach-Wolff et al., 2009), impaired insight (Erickson et al., 2011), cognitive dysfunction (Tsang, Leung, Chung, Bell, & Cheung, 2010), duration of untreated psychosis (Chang et al., 2012) and older age of schizophrenia onset (Ran et al., 2011), comorbid metabolic syndrome (Medeiros-Ferreira, Obiols, Navarro-Pastor, & Zuniga-Lagares, 2013) and the number (Lay, Blanz, Hartmann, & Schmidt, 2000) and duration (Nordt, Muller, Rossler, & Lauber, 2007) of hospitalizations. Finally, it has been found that agricultural or manual labour jobs, which are common in developing countries, may contribute to higher employment rates (Srinivasan, Rajkumar, & Padmavathi, 2001; Srinivasan & Thara, 1997; Srinivasan & Tirupati, 2005; Suresh et al., 2012).

To date, vocational status of schizophrenia patients in Middle and Eastern European countries was investigated only in the International Pilot Study of Schizophrenia (IPSS) (‘The International Pilot Study of Schizophrenia’, 1974; Sartorius, Shapiro, Kimura, & Barrett, 1972). This study estimated employment rates in schizophrenia patients from Prague and Moscow at 70% and 90%, respectively. However, the IPSS study was performed in the year 1968, and thus might not reflect current epidemiological status. Poland is considered to be one of the most rapidly developing post-communist countries in Middle and Eastern Europe. Eurostat estimated unemployment rate in Poland at 35.1% in people aged 20–64 years in 2013 (‘http://epp.eurostat.ec.europa.eu/tgm/table.do?tab=table&init=1&plugin=1&language=en&pcode=t2020_10’). Psychiatric health care has been changed significantly in Poland in the last few decades. Currently, psychiatric health care consists of inpatient units, day hospitals and outpatient clinics. Notably, public psychiatric setting constitutes the majority of the Polish mental health-care system. A gradual shift from inpatient to outpatient care was established as one of most important aims in the National Programme of Mental Health and is widely observed nowadays (‘National Programme of Mental Health’, 2010). This strategy promotes involvement in social environment facilitating functional recovery. Given that studies addressing predictors of employment in schizophrenia patients have provided mixed results and there is a lack of more recent studies investigating this issue in Middle and Eastern European countries, we performed a nationwide survey illustrating employment of schizophrenia patients in Poland. In this study, we aimed at calculating employment rates and indicating predictors of vocational status in this group of patients.

## Method

### Sampling

Data were gathered from public outpatient clinics between April and May 2013. Expected number of studied participants was 1,000 subjects. Assessment of outpatients’ medical records was performed in the cities, which were randomly selected in each from 16 Polish states established on the basis of an official administrative division. The probability of selecting each city was adjusted for the number of inhabitants. The inclusion criteria were as follows: (1) schizophrenia was diagnosed according to International Classification of Diseases, Tenth Revision (ICD-10) criteria at least 2 years prior to the day of assessment and (2) participants were aged over 18 years. Patients with comorbid neurologic disorders and substance use disorders, as well as schizoaffective disorder were excluded from the study. Participants gave written informed consent to participate in protocols approved by local Institutional Review Board. The study was performed in accordance with Declaration of Helsinki from the year 1964 and its later amendments, as well as the International Code on Market and Social Research developed joinly by the International Chamber of Commerce/European Society for Opinion and Market Research (ICC/ESOMAR). All psychiatrists participating in the study had at least 2-year experience in psychiatry, since it is obligatory to work at public outpatient clinics in Poland. Every psychiatrist was obliged to gather demographic and clinical data for the random sample of 10 schizophrenia outpatients meeting the above mentioned criteria. The list of patients included in each outpatient clinic was prepared by medical registrar, who was blinded to the purpose and methodology of the study. A total of 101 psychiatrists were approached in this study. The following data were collected: age, gender, education, learned profession, the number of visits in outpatient clinic within the last 12 months, age of schizophrenia diagnosis, information on previous hospitalizations, current vocational status and at the time of schizophrenia diagnosis, as well as the presence of physical health impairments (obesity, hypertension and diabetes). Data were collected based on medical history records, interviews with the patients and caregivers, as well as medical staff members. Following groups of patients were considered as employed: (1) subjects working in a full-time or part-time job; (2) patients who were self-employed (defined as earning income from own business or profession rather than by working for someone else); (3) those during work rehabilitation or sheltered work; (4) students; (5) patients who were retired due to age. Finally, 1,010 outpatients were randomly selected in all states. None of the selected outpatient clinics refused to participate in this study. However, the following data were not obtained for some participants: age (1 patient), gender (9 patients), education (9 patients), age of schizophrenia diagnosis (1 patient), mode of first hospitalization (83 patients), the number of years from a diagnosis time point (71 patients), employment status on the day of diagnosis (8 patients), employment status on the day of assessment (3 patients), the number of visits at outpatient clinics during last 12 months (10 patients), as well as information about comorbidities, including obesity (21 patients), diabetes (26 patients) and hypertension (22 patients).

### Statistics

Data were analysed using the Statistical Package for Social Sciences (SPSS) computer program. Employment was the dependent variable and was treated as a binary variable (employed vs. unemployed). Bivariate analyses were performed using chi-square for categorical data and *t*-test for continuous data. Mean and standard deviation (*SD*) were used to describe continuous variables. All variables significantly associated with maintaining vocational status were subsequently analysed using a binary logistic regression to test their independent significance. Kaplan–Meier survival analysis was used to assess the difference in the age of onset of schizophrenia between males and females. Differences between two survival curves were tested with log-rank test. Differences were considered as statistically significant if the *p* value was <.05. Due to multiple comparisons performed, Bonferroni correction was applied to the level of significance obtained in the *t*-test and chi-square test.

## Results

**Figure 1.**
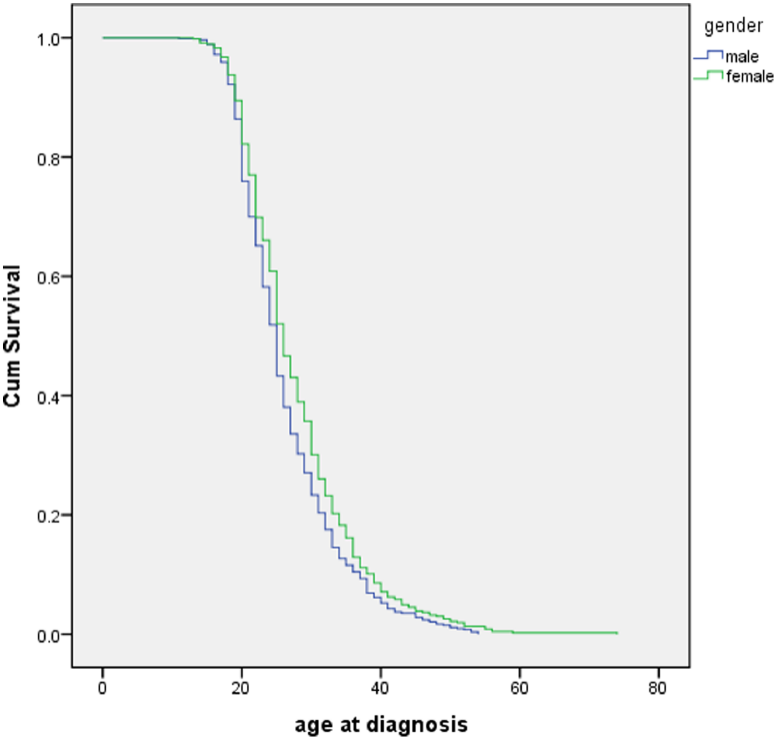
Age at diagnosis with respect to gender (536 males and 465 females, *p* = .02).

Table 1 presents general characteristics of studied participants. The mean age of schizophrenia patients was 38.48 ± 11.24 years. The majority of patients were males (53.59%) and had secondary or basic vocational education (36.76%). The most prevalent physical health comorbidity on the day of assessment was obesity (27.19%) that was followed by hypertension (13.76%) and diabetes (7.42%). Employment rates were estimated at 69.76% on the day of diagnosis and 25.82% on the day of assessment. The majority of employed schizophrenia patients were clerical workers (63.88%). Age at diagnosis was significantly lower in male patients in comparison with females (Figure 1).

**Table 1.** General characteristics of studied participants.

Patient characteristics	Cases/*N* (%)*	Mean ± *SD*
Age (years)		38.48 ± 11.24
Gender		
Males	536/1,001 (53.55%)	
Females	465/1,001 (46.45%)	
Education		
Higher	253/1,001 (25.27%)	
Other than higher	747/1,001 (74.73%)	
Employment on the day of diagnosis		
Employed	699/1,002 (69.76%)	
Unemployed	303/1,002 (30.24%)	
Employment on the day of assessment		
Employed	260/1,007 (25.82%)	
Unemployed	747/1,007 (74.18%)	
Prevalence of physical health impairments (on the day of assessment)		
Obesity	269/989 (27.19%)	
Hypertension	136/988 (13.76%)	
Diabetes	73/984 (7.42%)	
The number of visits at outpatient clinic within last 12 months		6.58 ± 3.66

*SD*: standard deviation.

* The number of cases per total number of patients, for whom particular data was available

Table 2 illustrates the comparison of two groups of patients: those who were employed at the time of being diagnosed and were unemployed on the day of assessment (SCZ(E–)), and patients who were employed at the time of being diagnosed and maintained employment activity until the day of assessment (SCZ(E+)). Patients from the SCZ(E+) group were significantly younger (*p* < .001) in comparison with those from the SCZ(E–) group (34.94 ± 9.94 years vs. 40.54 ± 11.69 years). Higher education was significantly more common among SCZ(E+) patients in comparison with SCZ(E–) subjects (28.13% vs. 17.03%, *p* = .001). There were also significant differences in vocational status on the day of diagnosis – SCZ(E+) patients were more commonly students, had higher rates of self-employment and lower rates of full employment when compared with SCZ(E–) individuals (*p* = .005). SCZ(E+) patients were living more frequently in middle- and high-income regions in comparison with SCZ(E–) subjects. Furthermore, the number of visits at outpatient clinics was significantly higher in SCZ(E–) than in SCZ(E+) patients (*p* = .043). Both groups differed considerably in terms of comorbid physical health impairments. SCZ(E–) patients were more likely to have hypertension (17.58% vs. 10.96%, *p* = .014), obesity (31.42% vs. 20.96%, *p* = .002) and diabetes (9.27% vs. 4.40%, *p* = .015), when compared with SCZ(E+) group. Illness duration measured as the time from the diagnosis time point to the day of assessment was significantly higher in SCZ(E–) subgroup than in SCZ(E+) patients (13.40 ± 9.78 years vs. 8.71 ± 7.62 years, *p* < .0001). In addition, first hospitalization at day care unit in comparison with inpatient unit predicted better vocational outcome throughout the course of schizophrenia (*p* = .027). Finally, total number of hospitalizations together with the number of inpatient hospitalizations were significantly higher in SCZ(E–) patients in comparison with SCZ(E+) patients (3.15 ± 1.56 vs. 1.92 ± 1.40, *p* < .0001 and 3.32 ± 2.96 vs. 1.6 ± 1.48, *p* < .0001, respectively). There were no between group differences with respect to gender (*p* = .089), age of diagnosis (*p* = .461) and the number of day hospitalizations (*p* = .594). After application of Bonferroni correction, differences in age, education, prevalence of obesity, number of years from the diagnosis time point, total number of hospitalizations and the number of inpatient hospitalizations remained significant (*p* < .0038).

**Table 2.** The comparison of schizophrenia patients, who were employed during the onset of psychosis and are currently unemployed (SCZ(E–)) with those who maintained vocational activity (SCZ(E+)) with respect to demographic and clinical variables.

Patient characteristics	SCZ(E+)	SCZ(E–)	*p*
Age	34.94 ± 9.94	40.54 ± 11.69	**<.0001^†^**
Gender			
Males	109/228 (47.81%)	255/465 (54.84%)	.089
Females	119/228 (52.19%)	210/465 (45.16%)	
Education			
Higher	63 (28.13%)	79 (17.03%)	**.001^†^**
Other than higher	161 (71.87%)	385 (82.97%)	
Vocational status on the day of diagnosis			
Student	93/230 (40.43%)	140/469 (29.85%)	**.005**
Full-time employee	102/230 (44.35%)	246/469 (52.45%)	
Self-employment	16/230 (6.96%)	20/469 (4.26%)	
Other forms of employment	19/230 (8.26%)	63/469 (13.44%)	
Region of residence^a^			
Low-income	61/230 (26.52%)	180/469 (38.38%)	**.008**
Middle-income	133/230 (57.83%)	225/469 (47.97%)	
High-income	36/230 (15.65%)	64/469 (13.64%)	
Number of consultations at outpatient clinic (last 12 months)	6.11 ± 3.08	6.87 ± 3.75	**.043**
Prevalence of physical health impairments			
Hypertension	25/228 (10.96%)	80/455 (17.58%)	**.014**
Obesity	48/229 (20.96%)	143/455 (31.42%)	**.002^†^**
Diabetes	10/227 (4.40%)	42/453 (9.27%)	**.015**
Number of years from the diagnosis time point	8.71 ± 7.62	13.40 ± 9.78	**<.0001^†^**
Age of diagnosis	26.23 ± 7.07	27.15 ± 7.40	.461
First hospitalization			
At day hospital	11/113 (9.73%)	12/312 (3.84%)	.027
At inpatient unit	102/113 (90.27%)	300/312 (96.16%)	
Total number of hospitalizations	2.00 ± 1.60	3.82 ± 3.32	**<.0001^†^**
The number of day hospitalizations	0.339 ± 0.69	0.379 ± 1.04	.594
The number of inpatient hospitalizations	1.6 ± 1.48	3.32 ± 2.96	**<.0001^†^**

Data expressed as the number of cases per the number of recorded patients for whom particular data were available (%) with exception of age, number of consultations at outpatient clinics, number of years from the diagnosis time point at outpatient clinics (during last 12 months) and numbers of hospitalizations (data expressed as mean value ± standard deviation (*SD*)).

Significant differences (*p* < .05) are marked in bold.

a Low-income regions: Warmia-Masuria, the Łódź province, the Świętokrzyskie province, Lesser Poland, Subcarpathia, the Lublin province, the Podlasie province; middle-income regions: Lower Silesia, the Lubusz province, West Pomerania, Pomerania, Greater Poland, the Opole province, Silesia, Kuyavia-Pmerania; high-income regions: Masovia.

† Significant differences after application of Bonferroni correction (*p* < .0038).

The logistic binary regression analysis (Table 3) confirmed significant differences between SCZ(E+) and SCZ(E-) patients in terms of education (*B* = −.152, odds ratio (OR) = 0.858, 95% confidence interval (CI): 0.745–0.988, *p* = .034), the number of consultations at outpatient clinics (*B* = .072, OR = 1.074, 95% CI: 1.011–1.141, *p* = .020), prevalence of obesity (*B* = −.513, OR = 0.599, 95% CI: 0.386–0.929, *p* = .022) and the number of inpatient hospitalizations (*B* = .434, OR = 1.534, 95% CI: 1.336–1.784, *p* < .001). These results imply that lower education level, higher number of consultations at outpatient clinics, obesity and higher number of hospitalizations were independently associated with the loss of employment in the course of schizophrenia.

**Table 3.** Predictors of vocational status in a logistic binary regression analysis.

Variables	*B*	OR (95% CI)	*p*
Age	.13	1.013 (0.983–1.044)	.409
Education	−.153	0.858 (0.745–0.988)	**.034**
First hospitalization (day or inpatient hospitalization)	−.037	0.964 (0.440–2.111)	.926
Vocational status on the day of diagnosis	.005	1.005 (0.910–1.109)	.925
Number of consultations at outpatient clinic	.072	1.074 (1.011–1.141)	**.020**
Obesity	−.513	0.599 (0.386–0.929)	**.022**
Region of residence	.099	1.104 (0.850–1.433)	.460
The number of inpatient hospitalizations	.434	1.544 (1.336–1.784)	**<.001**
Number of years from the diagnosis time point	.009	1.009 (0.973–1.047)	.620

*B*: beta; CI: confidence interval; OR: odds ratio.

Significant differences (*p* < .05) are marked in bold.

## Discussion

Given that public psychiatric care setting widely predominates private mental health facilities, this is the first representative study evaluating vocational status in schizophrenia patients in Poland after the collapse of communist regime. Furthermore, this is the only study performed in any of the Middle and Eastern European countries investigating clinical and demographic predictors of vocational status in this group of patients. Employment rate derived from our study was 25.82% and corresponds with estimates provided in the recent systematic review in this field (Marwaha & Johnson, 2004). However, it is still considerably lower in comparison to the general Polish population, in which employment rate has been estimated at 64.9% (‘http://epp.eurostat.ec.europa.eu/tgm/table.do?tab=table&init=1&plugin=1&language=en&pcode=t2020_10’). However, this employment gap between schizophrenia patients and the general population should be interpreted with caution as different definitions of employment were used by our group and Eurostat, that is, in contrast to our definition of employment, Eurostat included people aged 20–64 years and did not include subjects who were retired and students.

In agreement with previous studies, we have found that lower educational attainment and higher number of inpatient hospitalizations are associated with worse vocational outcome (Cougnard et al., 2009; Lay et al., 2000; Rogers, Anthony, Cohen, & Davies, 1997). However, it should be noted that lower education level is also linked with worse vocational outcome in the general, non-clinical population. In the recent study by Simone, Carolin, Max and Reinhold (2013), it was found that an increase of 1% in unemployment rate was associated with a 5% rise in admission rates for schizophrenia and an increase of 1% in working population was associated with a decrease of admission rates by 2%. These findings are also in line with studies showing that higher number of hospitalizations might predict less favourable functional outcome (Spellmann et al., 2012). However, we did not confirm such association for day hospitalizations. Indeed, it has been found that caring for patients at acute day hospitals might be as effective as treatment at inpatient units in terms of psychopathology, treatment satisfaction and subjective quality of life but more effective with respect to social disabilities (Kallert et al., 2007). These findings suggest beneficial effects of shifting psychiatric care into outpatient and day care setting, the phenomenon taking place in some countries (Rittmannsberger et al., 2004). According to Yearbooks of Mental Health published by the Institute of Psychiatry and Neurology in Warsaw that collects data from public psychiatric facilities, the number of day care places increased from 3,271 to 4,302, while the number of inpatient beds decreased from 17,286 to 16,174 in the years 2008–2012 (‘http://www.ipin.edu.pl/wordpress/IPiN_RS/search.html’).

We have also found that patients who lost employment in the course of schizophrenia visit outpatient clinics more frequently in comparison with patients who maintained vocational activity since a diagnosis time point. These findings might be due to more severe functional disability in patients with poor vocational outcome that requires more intense care and are in agreement with previous studies showing that steady employment is associated with significant reductions in mental health service use (Bush et al., 2009). However, it should be noted that obtained OR for the number of visits at outpatient clinics was relatively small suggesting that the effect of this variable, although significant, was not strong.

Most interestingly, we have found that physical health impairments might contribute to functional disability in terms of vocational activity. Multivariate regression analysis revealed that obesity is significantly more prevalent among patients who become unemployed in the course of schizophrenia in comparison with those who maintained employment. Metabolic syndrome and its components are highly prevalent among schizophrenia patients. The recent meta-analysis has estimated the prevalence of metabolic syndrome and obesity at 32.5% and 49.4%, respectively (Mitchell et al., 2013). To date, there is only one study that revealed the association between metabolic syndrome and inactive employment status (Medeiros-Ferreira et al., 2013). However, the link between obesity and employment activity has not been investigated so far, and a cross-sectional design of this study does not enable to draw conclusions on the direction of causality.

Our study has some limitations that should be addressed. First, this study was based on a cross-sectional design that does not allow inferring causal links. For instance, obesity was significantly more common among patients with poor vocational outcome. However, based on our data we cannot indicate the direction of causality. This study design is also important with respect to medical comorbidities that are often underdiagnosed among schizophrenia patients (Barnes, Paton, Cavanagh, Hancock, & Taylor, 2007; Konz et al., 2014), and the lack of objective case identification based on clinical and biochemical measures might have led to underestimation of prevalence rates. It is also important that this study design does not allow following dynamic changes in vocational status. Another limitation is the lack of some important demographic and clinical variables, such as marital status and rural or urban place of residence, type and dosage of medication regimen, the number of hours the patients worked as well as measures of psychopathology and cognitive functioning that have been repeatedly found to influence employment status in schizophrenia patients. Finally, some data were not available for all selected participants including age, gender, education, age of schizophrenia diagnosis, mode of first hospitalization, the number of years from a diagnosis time point, employment status on the day of diagnosis, employment status on the day of assessment, the number of visits at outpatient clinics during last 12 months, as well as information about comorbidities. However, the number of participants with missing data was relatively low.

## Conclusion

This study suggests that education level, the number of inpatient hospitalizations and obesity are strong predictors of vocational outcome in schizophrenia. Future studies should indicate the direction of causality between vocational impairment and medical comorbidities. Similarly, it is unclear as to whether higher number of inpatient hospitalizations simply reflects highly relapsing course of schizophrenia and thus contributes to poor employment status or reflects lower efficacy of inpatient treatment with respect to vocational disabilities that may require additional psychosocial interventions. Results of this study may imply that interventions aimed at reducing the number of inpatient hospitalizations and improving physical health as well as social support strategies may contribute to better vocational outcomes.
